# Comments and Reply to: Foot Plantar Pressure Measurement System: A Review. *Sensors* 2012, *12*, 9884-9912

**DOI:** 10.3390/s130303527

**Published:** 2013-03-13

**Authors:** Nachiappan Chockalingam, Aoife Healy, Roozbeh Naemi, Philip Burgess-Walker, Abdul Hadi Abdul Razak, Aladin Zayegh, Rezaul K. Begg, Yufridin Wahab

**Affiliations:** 1 Centre for Sport, Health and Exercise Research, Faculty of Health Sciences, Staffordshire University, ST4 2DF, UK; E-Mails: a.healy@staffs.ac.uk (A.H.); r.naemi@staffs.ac.uk (R.N.); p.burgess-walker@staffs.ac.uk (P.B.-W.); 2 School of Engineering and Science, Victoria University, Melbourne 3000, Australia; E-Mail: Aladin.Zayegh@vu.edu.au; 3 Faculty of Electrical Engineering, Universiti Teknologi MARA, Shah Alam 40000, Selangor, Malaysia; 4 Institute of Sport, Exercise and Active Living (ISEAL) and School of Sport and Exercise Science (SES), Victoria University, Melbourne 3000, Australia; E-Mail: Rezaul.Begg@vu.edu.au; 5 Centre for Industrial Collaboration, School of Microelectronic Engineering, Universiti Malaysia Arau, Perlis 02600, Malaysia; E-Mail: yufridin@unimap.edu.my

## Comments

We would like to comment on a recent review article published in Sensors by Razak *et al.* [1]. The authors provided a review of plantar pressure measurement systems which included the discussion of the recently developed WalkinSense^®^ system. While the authors correctly identified that our group completed research using this system [2], they have inaccurately reported the manufacturer of the system and our research findings.

The authors stated that this system was developed by our group, however, as stated in our article [2], this system was developed by Tomorrow Options Microelectronics (Sheffield, UK). Additionally they stated inaccurately that the WalkinSense^®^ system is similar to the F-Scan^®^ (Tekscan, Boston, MA, USA) hardware and software and that our research concluded that WalkinSense^®^ had a better repeatability when compared to other commercially available systems. Our research [2] clearly concluded that the WalkinSense^®^ system was found to be as repeatable as the F-Scan^®^ system.

There are several papers which outline the existing technologies [3], the protocols used for data collection [4,5] and the repeatability of existing systems [6]. Whilst this paper [1], is mainly intended for a different audience when compared to clinical papers, it is very important that previously published information is carefully considered, critically reviewed and succinctly presented in a review paper.

We felt compelled to write this note so that: (1) it is not misinterpreted that we developed this system and (2) our research findings are not inaccurately reported.

Nachiappan Chockalingam Aoife Healy Roozbeh Naemi Philip Burgess-Walker
