# Supplementary material for: Comments and Reply to: Foot Plantar Pressure Measurement System: A Review. Sensors 2012, 12, 9884-9912
Source: Sensors (Basel). 2013 Mar 13;13(3):3527–9. doi: 10.3390/s130303527 (PMC3658759; doi:10.3390/s130303527)
Supplement: Related texts

# Reply

**Published:** 2013-03-13

We acknowledge your concerns and would like to apologize for the unintended errors in our paper [1] while quoting two points from Healy *et al.* [2]. Firstly, the manufacturer of the system is inaccurately identified. Secondly, our sentence “has a better repeatability compared to other commercially available systems” should rather read “equal repeatability compared to F-Scan system”.

The corrections made are as listed below:

- On page 9896, lines 12–13, the sentence “has a better repeatability compared to other commercially available systems” should be “equal repeatability compared to the F-Scan^®^ system”.
- On page 9897, lines 2–3, the sentence “It appears that only the sensor is their own development, the rest of the system is similar to F-Scan^®^ (Tekscan, USA) hardware and software.” should be “The “WalkinSense” system was developed by Tomorrow Options Microelectronics (Sheffield, UK). This portable system's hardware includes eight individual sensors as well as a data acquisition and processing unit, whereas the proprietary software (WalkinSense version 0.96) records and displays plantar pressure profiles”.
- On page 9897, line 14, the sentence “developed their own transducer but seems to have a limited number of sensor placements” should be “has a limited number of sensor placements”.
- On page 9900, line 24, the sentence “used F-Scan^®^ (Tekscan, USA) for the sensor” should be “used a low-cost pressure transducer”.

Abdul Hadi Abdul Razak Aladin Zayegh Rezaul K. Begg Yufridin Wahab

## References

[1] Abdul Razak A.H., Zayegh A., Begg R.K., Wahab Y. (2012). Foot Plantar Pressure Measurement System: A Review. Sensors.

[2] Healy A., Burgess-Walker P., Naemi R., Chockalingam N. (2012). Repeatability of WalkinSense^®^ in Shoe Pressure Measurement System: A Preliminary Study. The Foot.
